# Osseointegration aspects of placed implant in bone reconstruction with newly developed block-type interconnected porous calcium hydroxyapatite

**DOI:** 10.1590/1678-775720150597

**Published:** 2016

**Authors:** Kazuya DOI, Takayasu KUBO, Yusuke MAKIHARA, Hiroshi OUE, Koji MORITA, Yoshifumi OKI, Shiho KAJIHARA, Kazuhiro TSUGA

**Affiliations:** Hiroshima University Graduate School of Biomedical and Health Sciences

**Keywords:** Implant, Hydroxyapatite, Bone regeneration

## Abstract

Artificial bone has been employed to reconstruct bone defects. However, only few reports on implant placement after block bone grafting exist.

**Objectives:**

The purpose of this study was to evaluate the osseointegration of dental implant in bone reconstructions with interconnected porous calcium hydroxyapatite (IP-CHA).

**Material and Methods:**

The IP-CHA cylinders (D; 4.3 mm, H; 10.0 mm) were placed into bone sockets in each side of the femurs of four male dogs. The IP-CHA on the right side was a 24-week sample. Twelve weeks after placement, a titanium implant was placed into a socket that was prepared in half of the placed IP-CHA cylinder on the right side. On the left side, another IP-CHA cylinder was placed as a 12-week sample. After another 12 weeks, the samples were harvested, and the bone regeneration and bone-implant contact (BIC) ratios were measured.

**Results:**

New bone formation area was superior in the 24-week IP-CHA compared with the 12-week IP-CHA. BIC was not significantly different between IP-CHA and the parent sites. Osseointegration was detected around the implant in IP-CHA-reconstructed bone.

**Conclusion:**

Our preliminary results suggest that IP-CHA may be a suitable bone graft material for reconstructing bones that require implant placement.

## INTRODUCTION

Bone reconstruction in combination with bone grafting is used at sites with insufficient bone for proper implant placement. However, dental implant placement using guided bone regeneration (GBR) is quite difficult with large bone defects caused by trauma, tumors, or severe periodontal disease. In such cases, implant placement is performed after bone reconstruction using bone grafting^19,27^. De Santis, et al.^6^ (2012) evaluated implant placement into contemporaneous mandibular defects. In that study, the implant and autologous bone were simultaneously placed on one side, while another implant was placed on the other side following autologous block bone grafting (delayed implant placement). The bone-to-implant contact ratio (BIC) in the delayed implant placement was higher than that in the simultaneous implant and autologous bone block placement^6^. This suggests that implant placement after preliminary bone reconstruction would be suitable for GBR of large defects. Considering graft material shape, the granular type of artificial bone used in GBR is difficult to apply to large bone defects because of poor mechanical strength and retention morphology^11,29^.

Therefore, preliminary bone reconstruction for implant placement requires a block-type material with high biocompatibility and good osteoconduction. Block-type bone graft materials are also used as autologous calvarias or iliac crest bone blocks before implant placement^9,15,26,27^. The beneficial outcomes of implant placement into grafted sites with autologous bone blocks have been described^6,9,28^. Unfortunately, autologous bone grafting can be problematic because the harvest may not yield sufficient bone for grafting, which can cause persistent pain, nerve damage, fracture, or cosmetic defects at the donor site^4,6,20^. Recently, interconnected porous calcium hydroxyapatite (IP-CHA) was introduced as a novel biomaterial for bone regeneration^25^ and is now widely used in both clinical and experimental fields^7,8,13,22,24^. Because IP-CHA comprises a systematic arrangement of uniform, spherical, interconnecting pores, it can provide favorable scaffolding, allowing cells or agents’ access into the internal structures. In our previous animal studies, granular IP-CHA was used in mandibular bone defects and fenestrated defects around the implants, and the results indicated superior bone regeneration and osseointegration^7,13^. The block-type IP-CHA also exhibited favorable osteoconduction, with regenerated bone detected in both the superficial and deep portions of the IP-CHA^18,30^. These findings indicate that IP-CHA-reconstructed sites may be undergoing bone remodeling in the parent bone tissue. Therefore, it is expect from bone reconstruction sites with IP-CHA to achieve osseointegration after implant placement.

The purpose of this study was to evaluate the osseointegration of implants placed in sites reconstructed with IP-CHA blocks.

## MATERIAL AND METHODS

### Material

**Figure 1:**
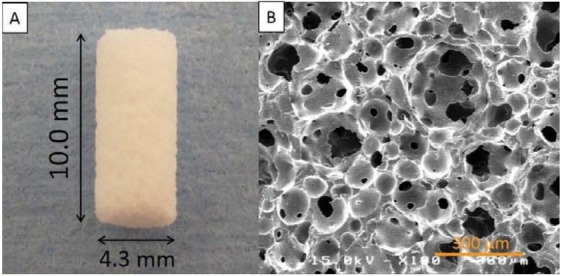
IP-CHA structure. (A) Photograph of prepared block-type IP-CHA cylinder; (B) A scanning electron microscope image of the IP-CHA surface. IP-CHA has a systematic arrangement of uniform pores, all of which are connected by a network of smaller interconnected pores

IP-CHA cylinder blocks (diameter; 4.3 mm, height; 10.0 mm (Covalent Materials, Tokyo, Japan) were fabricated for this study. This artificial bone has 75% porosity and a mean pore diameter of 150 µm (all pores were interconnected with 40 µm diameter pores) (Figure 1). IP-CHA was manufactured using the ‘‘form-gel’’ technique^25^. Pure titanium implants were also used (diameter; 3.3 mm, length; 10.0 mm, Brånemark System Tiunite^TM^ Mk III, Nobel Biocare, Kloten, Switzerland).

### Animals and surgical procedures

The animal research protocol was in accordance with the current version of the Japan Law on the Protection of Animals. This study was approved by the Research Facilities Committee for Laboratory Animal Science at the Hiroshima University School of Medicine, Hiroshima, Japan (Approved No. A11-98).

All the surgeries were performed under general anesthesia with sodium pentobarbital (10 mg/kg) and local infiltration anesthesia with 2% lidocaine and 1:80,000 noradrenaline. Every effort was made to minimize animal suffering during the experimental period.

**Figure 2:**
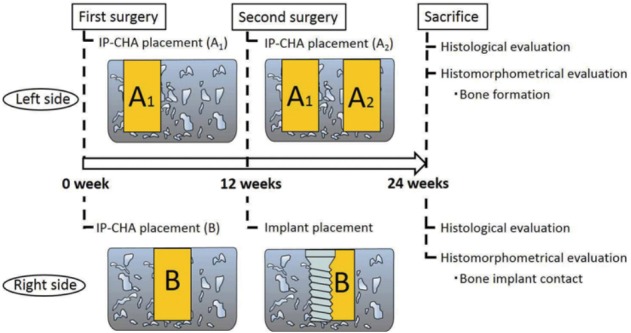
Animal experiment design

The study time line is shown in Figure 2. The study was performed in two phases. On the left side, we evaluated bone healing or formation with the IP-CHA block 12 and 24 weeks after placement. For the right femur, we evaluated dental implant osseointegration with the previously placed IP-CHA at 12 weeks compared with the side connected to the femoral cortical bone.

The experimental subjects were four male Beagle-Labrador hybrid dogs (weight; 20-23 kg, age; 18-20 months).

**Figure 3:**
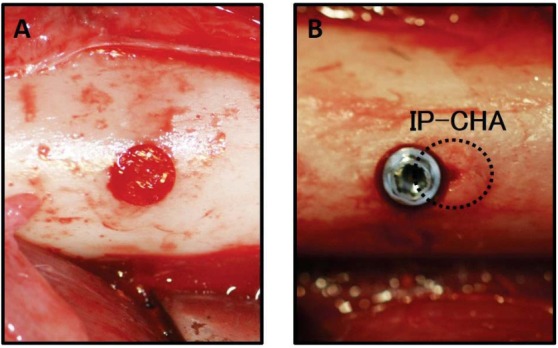
Right femur image. (A) IP-CHA placement into a bone socket in the right femur; (B) The implant was placed in the grafted IP-CHA, with half of the implant contacting the IP-CHA surface. The dotted line indicates previously grafted IP-CHA

The animals were fed in their cages for one month to allow acclimatization. During the first surgery, each IP-CHA cylinder block was placed into pre-prepared bone sockets in each side of the femur (left; sample A1, right; sample B, Figure 3A). Twelve weeks after the first surgery, a second surgery was performed. On the left side, a second IP-CHA block was placed adjacent to the original IP-CHA block (sample A1) as a 12-week sample (sample A2). On the right side, sockets were prepared in the central portion of the grafted IP-CHA and beside the parent bone site in the femur, and the implant was then placed into the socket (Figure 3B). Consequently, half of the implant was in contact with the previously placed IP-CHA block, while the other half was in the femur bone. Implant socket preparation was performed using a special power tool with serial cutting drills and a screw tap in accordance with the Brånemark system^®^ manual. To minimize bone damage, we used low-speed and low-pressure drilling and continuous external saline irrigation. Twelve weeks after the second surgery, the animals were sacrificed, and the bone tissue blocks containing IP-CHA and/or implant were obtained.

### Specimen preparation

All tissue blocks were fixed in 10% neutral formalin. The ones without implant (samples A1 and A2) were decalcified using K-CX^®^ solution (FALMA, Tokyo, Japan) for one week. The blocks were then dehydrated through a graded ethanol series, cleared in xylene, and embedded in paraffin. Sections with 5 µm thickness were obtained and stained with hematoxylin and eosin. Tissue blocks with implant (sample B) were dehydrated using ascending concentrations of ethanol, cleared with styrene monomer, and embedded in light-polymerized polyester resin (Technovit 7200VLC, Heraeus Kulzer, Wehrheim, Germany). Photo-polymerization equipment was used (BS5000, EXAKT Apparatebau, Norderstedt, Germany) to ensure complete polymerization before the specimens were sectioned with a high-precision diamond disc to produce 200 µm thick cross-sections. Undecalcified specimens were ground to approximately 70 µm thickness with a special grinding machine. (MG5000, EXAKT Apparatebau, Chemnitz, Germany) and stained with toluidine blue. New bone formation and BIC were evaluated histologically and histomorphometrically.

### Histomorphometric evaluation

**Figure 4:**
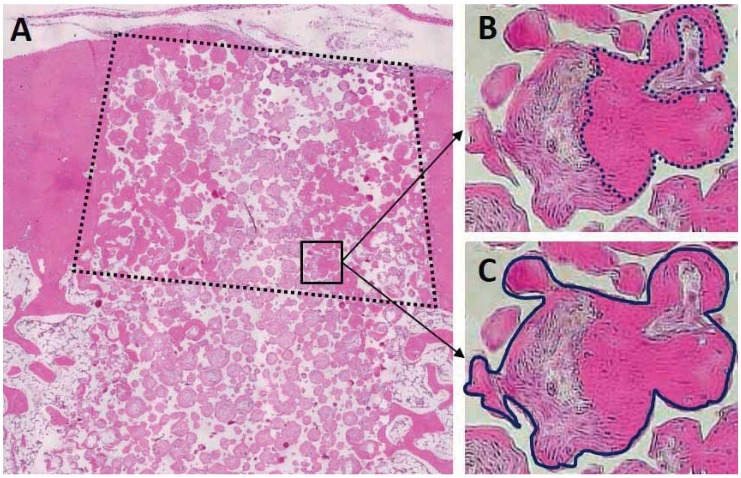
Schematic drawing of hstomorphometric analysis. The ratio of bone formation was measured in the cortical bone area (A, dashed box). It was calculated as the ratio of the area of newly formed bone (B, inside the dotted line) to that of the total regenerated tissue (C, inside the solid line)

New bone formation area was measured on samples A2 (12-week) and A1 (24-week). Newly formed bone in the IP-CHA pores at the cortical area was quantified as the ratio of generated bone to the total cortical bone area (Figure 4) (ImageJ software, National Institutes of Health, Bethesda, MD, USA). The central portion of each section was measured.

The BIC was measured as well as the percentage of bone length in direct contact with the implant surface on the left side of the implant using ImageJ software. It was determined as the length of newly formed bone between the top and the bottom of the implant shoulder.

### Statistical analysis

Data are expressed as means ± standard deviations. The ratios of the new bone formation area and BIC values were statistically analyzed at the 5% significance level using Mann-Whitney U tests (n=4).

## RESULTS

### Bone formation evaluation of pre-prepared bone sockets grafted with IP-CHA block

**Figure 5:**
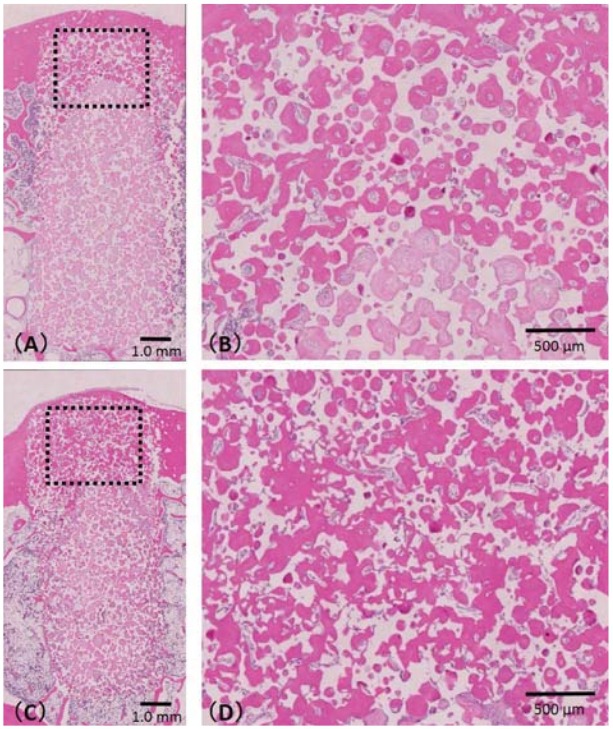
Histological specimen of samples A1 and A2. A1 (A) and A2 (C) are the 24- and 12-week samples, respectively. Newly formed bone was detected in the pores of both A1 and A2. In the center of the cortical bone area (in the dashed box), significantly more bone had been formed in the pores of (D) A1 compared with (B) A2

Figure 4 shows the samples A1 (Figure 5CD) and A2 (Figure 5AB). Newly formed bone was detected in the pores of both 12- and 24-week samples. In the center of the cortical bone area, bone and connective tissue were found in the 12-week IP-CHA (Figure 4B), but significantly more bone had been formed in the pores of the 24-week IP-CHA (Figure 4D).

**Figure 6:**
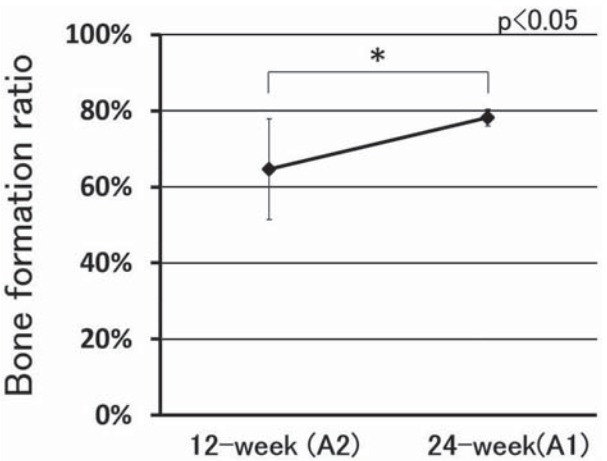
Ratio of newly bone formed of sample A. Asterisk indicates a significant difference between 12 and 24 weeks (p<0.05)

The ratio of new bone formation was 64.6±13.2% for the 12-week IP-CHA and 78.2±2.2% for the 24-week IP-CHA (Figure 6), showing a significantly higher value (p<0.05).

### Evaluation of osseointegration implants placed in sites reconstructed with IP-CHA

**Figure 7:**
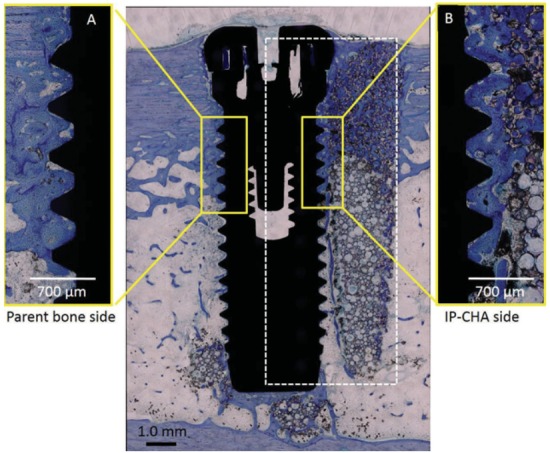
Histological specimen of sample B. (A) High magnification view of the implant surface at the parent bone site. New bone formation from preexisting cortical bone was detected, showing that osseointegration was achieved; (B) High magnification of the implant surface at the IP-CHA site. New bone formed in the IP-CHA pores in contact with the implant surface, showing that osseointegration was achieved. The bottom portion of the IP-CHA pores contained small amounts of new bone. (The dotted line indicates placed IP-CHA)

Figure 5 shows sample B with the right side implant. New bone formation from preexisting cortical bone was detected at the IP-CHA site and on the implant surface within the cortical bone area. New bone was formed in the pores of the IP-CHA block and parent bone sites. The formed bone could be observed in the interface between the bone and the implant thread, and osseointegration occurred on both sides (Figure 7A-B).

Bone resorption was not observed in the shoulder of the implant for either site (IP-CHA or parent bone). The mean of BIC at the grafted IP-CHA site (34.7±7.2%) and parent bone site (32.5±7.8%) were not significantly different (n=4, p=0.696) (Table 1).

**Table 1:** The rate of bone-implant contact (BIC)

(n=4)	BIC% (SD)
IP-CHA site	34.7 (7.2)
Parent bone site	32.5 (7.8)

SD=standard deviation

p=0.696

## DISCUSSION

This study demonstrates that osseointegration can be achieved in bone reconstructed using IP-CHA blocks; notably, BIC and integration were equivalent to those observed in the parent bone site.

Several studies have reported implant placement in bone-grafted sites using granular graft materials^3,5,10,16,28^. In dogs with mandibular defects, an implant placement into a grafted site reconstructed with bovine cancellous bone particles showed osseointegration with the newly formed surrounding bone^3^. Clinically, deproteinized bovine bone mineral (DBBM) and hydroxyapatite (HA) substitutes have been suggested as suitable graft materials for alveolar ridge preservation of extraction sockets to ensure optimal implant placement^5,16^. Generally, optimal particle size is considered between 300 and 600 µm; this diameter allows efficient migration of bone-producing cells and colonization by blood vessels, both of which are essential for new bone formation^12^. Through bone tissue formation and vascularization, the grafted site facilitates osseointegration. For these reasons, granular materials are frequently applied for bone grafting. However, they are difficult to use for large bone defects that are unlikely to be supported by surrounding bone, and granular materials lack the capacity to maintain their shape or mechanical stress. Micro movements of the grafted site during healing disturb bone generation but induce soft tissue formation around the grafted granules^11,29^. Minimizing mechanical stress, micro movement supports bone healing, making block-type graft materials suitable for reconstructing large bone defects. In this study, implants were placed into IP-CHA blocks after bone reconstruction instead of being simultaneously placed at the site with defect creation.

Although autologous bone from the jaw or iliac crest provides sufficient bone block volume for reconstruction, other problems limit the use of the procedure. In addition, autologous bone grafts may be resorbed at the grafted site, leading to insufficient bone volume to support the implant, compromising its optimal position.

Synthetic biomaterials are ideal for grafting because there are no risks from harvest limitation, donor innovation, or unforeseen infection. Although HA has been used for grafting, the traditional HA block is not suitable for bone reconstruction in implantation because of its dense structure and low porosity. It is widely accepted that because of the nucleus size in most mammalian cells, which is more than 10 µm, pore sizes greater than 10 µm in diameter permit osteoconduction^23^. Because of the low porosity of HA, ingrowth of bone-forming cells and vascularization from the recipient site was limited^2^. Bone ingrowth by HA with no interconnected structure was less than 300 µm at 4 months after implantation^1^. In addition, because of the dense structures and high mechanical strength, it is difficult to drill for the implant socket preparation. Therefore, preliminary implant placement with HA blocks is considered problematic. In contrast, the compressive mechanical strength of IP-CHA is approximately 10 Mpa, similar to that of the cancellous bone, and it gradually increases after placement in the bone because of its ingrowth into the pores. The degree of mechanical strength increased 3-fold three weeks after the implantation in a rabbit study^25^. In this study, implant sockets were easily created at the grafted IP-CHA site without excessive generation of frictional heat, and implants achieved primary stabilization. Through the interconnection of pores in IP-CHA, as described in the Material and Methods section, cell ingrowth and vascularization are possible with this material. The bone strength and density of implanted IP-CHA blocks increase over time due to osteoconduction^25^. Furthermore, clinical orthopedic results have shown that increasing bone strength with IP-CHA blocks can reduce the risk of bone fracture^14^.

We found that the bone formation ratio at 24 weeks was greater than that at 12 weeks, indicating that it continuously progressed in the grafted IP-CHA site. Histological observations confirmed newly formed bone at the implant surface of cortical bone area for both the reconstructed IP-CHA and parent bone sites. These findings indicate that grafted IP-CHA allows bone remodeling, and implants can therefore achieve osseointegration when bone reconstruction is performed with IP-CHA blocks.

Successful healing outcomes have also been described for DBBM blocks^10,21^. However, one report stated that DBBM blocks did not promote osseointegration more efficiently than the preexisting bone^9^.

A canine mandibular model demonstrated new bone formation, and all DBBM and autologous bone blocks were well integrated with the parent bone. However, the authors reported that both, as well as the bone formation area were significantly lower for DBBM than for the autologous bone block^9^. In this study, the BIC ratios were not significantly different between the IP-CHA and the parent bone sites, and osseointegration was detected in both conditions. In the case of implant placement into a site that was previously grafted with IP-CHA, it is considered that the implant is first mechanically stabilized by the surrounding bone tissues, and the level of stabilization gradually increases as newly formed bone integrates with the implant. This probably occurs because osteoconduction into IP-CHA from the surrounding bone generates an interconnected structure. Therefore, bone remodeling occurred around the implant at the preliminary grafted site with IP-CHA.

In our previous study, we placed the implant/IP-CHA complex in dog femurs. After 2 months, there was poor implant stability; however, the 3-month samples showed favorable implant stability and appropriate implant placement at parent bone site^9^. In addition, when the IP-CHA block and implant were simultaneously inserted in rabbit femoral condyles, implant stability was superior than that in parent bone site. These findings suggest that bone formation occurred in both the upper and lower portions because the interior of femoral condyle is largely cancellous^17^.

IP-CHA is a favorable grafting material for preliminary bone reconstruction before implant placement. However, it must be noted that this study was limited to an internal femoral defect, which is a favorable new bone formation environment. We intend to conduct further microtomographic studies on an onlay-type model or the mandible to investigate osseointegration and bone aspects of the IP-CHA block in greater detail.

## CONCLUSIONS

Our results indicate that a placed implant could achieve osseointegration in grafted IP-CHA sites as well as in parent bone sites. Based on these limited results, we suggest that IP-CHA blocks might be a useful bone substitute for bone reconstruction during simultaneous implant placement.
